# Peptides from the croceine croaker (*Larimichthys crocea*) swim bladder attenuate busulfan-induced oligoasthenospermia in mice

**DOI:** 10.1080/13880209.2022.2034895

**Published:** 2022-02-11

**Authors:** Xi Zhao, Mengmeng Sang, Ping Han, Jie Gao, Zhenhua Liu, Hu Li, Yayun Gu, Chengniu Wang, Fei Sun

**Affiliations:** Institute of Reproductive Medicine, Medical School, Nantong University, Nantong, Jiangsu Province, China

**Keywords:** Swim bladder peptides, Lin28b, Igf2bp1, PGK2, Cfap69

## Abstract

**Context:**

The swim bladder of the croceine croaker is believed to have a therapeutic effect on various diseases. However, there is no research about its effect on mammalian spermatogenesis.

**Objective:**

We investigated the swim bladder peptides (SBPs) effect on busulfan-induced oligoasthenospermia in mice.

**Materials and methods:**

We first extracted SBP from protein hydrolysate of the croceine croaker swim bladder, and then five groups of ICR male mice were randomly assigned: control, control + SBP 60 mg/kg, busulfan, busulfan + SBP 30 mg/kg and busulfan + SBP 60 mg/kg. Mice received bilateral intratesticular injections of busulfan to establish oligoasthenospermia model. After treatment with SBP for 4 weeks, testis and epididymis were collected from all mice for further analysis.

**Results:**

After treatment with SBP 30–60 mg/kg for 4 weeks, epididymal sperm concentration and motility increased by 3.9–9.6- and 1.9–2.4-fold than those of oligoasthenospermia mice induced by busulfan. Meanwhile, histology showed that spermatogenic cells decreased, leading to increased lumen diameters and vacuolization in the busulfan group. These features were reversed by SBP treatment. RNA-sequencing analysis revealed that, compared with the busulfan group, Lin28b and Igf2bp1 expression related to germ cell proliferation, increased with a >1.5-fold change after SBP treatment. Additionally, PGK2 and Cfap69 mRNAs associated with sperm motility, also increased with a >1.5-fold change. Furthermore, these findings were validated by quantitative real-time PCR and Western blotting.

**Discussion and conclusions:**

This is the first reported evidence for the therapeutic effect of SBP on oligoasthenospermia. SBP may be a promising drug for oligoasthenospermia in humans.

## Introduction

Infertility is a serious health problem worldwide. It is reported that 15% of couples suffer from fertility problems, half of which are caused by male factors (Wong and Cheng 2011; Levine et al. 2017). Impairments in spermatogenesis, such as oligoasthenospermia, are the main causes of male infertility. Busulfan is widely used as an anticancer chemotherapeutic agent in the treatment of chronic myelogenous leukaemia in children and adults. However, the extensive use of busulfan in clinic impairs testicular tissue and disrupts the process of spermatogenesis, resulting in oligoasthenospermia in male patients (Meistrich 2009; Brignardello et al. 2016). Therefore, it is urgent to search for therapies that can reduce these side effects of busulfan on male infertility.

Teleost swim bladders are rich in proteins, microelements, polysaccharides and vitamins. Traditional Chinese medicine practitioners assert that extracts from the swim bladder from the croceine croaker (*Larimichthys crocea* Richardson [Sciaenidae]) have good therapeutic effects on many diseases, including curing dizziness, protecting liver function and removing free radicals (Suo et al. 2015; Zhao et al. 2015). Furthermore, a peptide extracted from swim bladder proteins was reported to exhibit strong antioxidant and antifatigue activities (Zhao et al. 2016; Pal and Suresh 2017; Zheng et al. 2020). However, there has been no report on the effect of swim bladder peptides (SBPs) on mammalian spermatogenesis.

As is well known, a large number of genes are required for spermatogenesis. For example, Lin28b is recognized as central to coordinate proliferation and growth at the cellular level. This gene is expressed in spermatogonia and spermatocytes but not in spermatids or mature spermatozoa (Aeckerle et al. 2012; Sangiao-Alvarellos et al. 2015). The gene encoding insulin-like growth factor 2 mRNA-binding protein 1 (Igf2bp1) is also expressed mainly in spermatogonia and has been considered to play important roles in cell proliferation and in the growth of normal and tumorous tissues (Hammer et al. 2005; Huang et al. 2018; Zhang et al. 2020). In brief, Lin28b and Igf2bp1 are important in germ cell development.

Additionally, phosphoglycerate kinase 2 (PGK2) is encoded by an autosomal gene that is expressed exclusively during spermatogenesis and is important for sperm activity (Danshina et al. 2010; Liu et al. 2019). Cilia and flagella associated protein 69 (Cfap69) is located in the midpiece of the sperm flagellum, and its deletion causes a variety of abnormalities in the flagella in human and mouse spermatozoa, leading to male infertility (Dong et al. 2018; He et al. 2019; Mbango et al. 2019). PGK2 and Cfap69 proteins are closely related to sperm motility.

In the present study, we found that SBPs significantly increased sperm concentration and motility in mice with busulfan-induced oligoasthenospermia. Moreover, SBP could also increase the protein levels of Lin28b, Igf2bp1, PGK2 and Cfap69. These findings suggest that SBP promotes germ cell proliferation to increase sperm concentration via upregulation of Lin28b and Igf2bp1 proteins, and increases sperm motility via upregulation of PGK2 and Cfap69 proteins.

## Materials and methods

### Chemicals and reagents

Busulfan was purchased from Sigma-Aldrich (Darmstadt, Germany). TB Green™ Premix Ex Taq™ II was obtained from TakaRa Biotechnology (Dalian, China). Primary antibodies against Lin28b (24017-1-AP) and Igf2bp1 (22803-1-AP) were obtained from Proteintech (Rosemont, IL). Primary antibodies against PGK2 (ab183031), GAPDH (ab181602) and β-actin (ab8227) antibodies were purchased from Abcam (Cambridge, UK). Primary antibody against Cfap69 (30352) was purchased from Signalway Antibody (College Park, MD). Goat anti-rabbit IgG H&L (ab175781) antibody was purchased from Abcam (Cambridge, UK).

### Peptides preparation and purification

The swim bladders of croceine croakers were obtained from School of Food and Pharmacy, Zhejiang Ocean University. The swim bladders were soaked in 0.1 M NaOH to remove non-collagenous proteins according to the method of Zhao et al. (2016). The residues were washed, and then immersed in 10% butanol for 12 h, changing the solution every 3 h. The defatted samples were dispersed with 1:3 solid/solvent phosphate-buffered saline (PBS) and hydrolysed with alcalase at pH 9.0 for 8 h, and 45 °C. The protein hydrolysate was centrifuged, and the obtained supernatant was freeze-dried and named as SBPs.

### Animals and treatment

Male Institute of Cancer Research (ICR) strain mice (8 weeks old), weighting 30 ± 2 g, were obtained from the Experimental Animal Center of Nantong University (Nantong, China). All experimental procedures involving animals conformed to the National Institutes of Health guide for the care and use of Laboratory animals. The study was approved by the Institutional Animal Ethics Committee of Nantong University (permission no. S20190320-022).

Mice were randomly divided into five groups (*n* = 6): control, control + SBP 60 mg/kg, busulfan, busulfan + SBP 30 mg/kg and busulfan + SBP 60 mg/kg. Mice were anaesthetized and then received bilateral intratesticular injections of busulfan at a dose of 4 mg/kg body weight (Qin et al. 2016). Two weeks later, the control and busulfan-treated mice were given SBP at 30 or 60 mg/kg by gavage for 4 weeks. Finally, mice were sacrificed by being anaesthetized, and the testes and epididymis were collected from all mice for further detection.

### Testicular weight index, sperm concentration and motility

Mice were anaesthetized, and the mouse weight and testicular weight were determined at the end of the experiment. The testicular weight index was calculated as testicular wet weight (mg)/body weight (g)×100%.

Meanwhile, the epididymides from mice were removed while under anaesthesia, and the cauda epididymidis was incubated in warm PBS at 37 °C for 20 min and gently shaken to allow the spermatozoa to swim out. The suspensions of 200 μL each in 2 mL Tyrode’s solution were diluted. Diluted sperm suspension was put on a glass slide, and then sperm concentration and viability were calculated using a computer-assisted sperm analysis (CASA) system.

### Histology

Testes from each group were fixed overnight in 4% paraformaldehyde, and then dehydrated through a graded ethanol–water series, and embedded in paraffin wax. Sections were cut into 8 μm thickness, dewaxed and stained with haematoxylin and eosin (HE). Finally, the slides were then observed under a light microscope.

### RNA sequencing and data analysis

Total RNA was extracted from frozen-thawed testes with TRIzol reagent according to the manufacturer’s instructions (Thermo Fisher Scientific, Waltham, MA). It was fragmented into small pieces, and cDNA was produced by reverse transcription using random hexamer primers. Subsequently, the synthesized cDNA was terminally repaired and then 3′ adenylated. Polymerase chain reaction (PCR) products were purified with Ampure XP Beads (Agencourt Bioscience, Brea, CA), and then dissolved in EB solution. The library was validated using a 2100 bioanalyser (Agilent Technologies, Santa Clara, CA). The double-stranded PCR products were heat denatured and cyclized by splint oligo sequence method. Single strand circular DNA sequences were formatted as the final library. The library was amplified with phi29 DNA polymerase to generate DNA nanoballs (DNBs) each with more than 300 copies. The DNBs were loaded into a patterned nanoarray and a combined Probe-Anchor Synthesis was used to generate a single-end 50 (opposed-end 100/150) base reading. Finally, the differentially expressed genes were obtained via DEseq2 software (https://bioconductor.org/packages/release/bioc/html/DESeq2.html) and gene enrichment analysis against Gene Ontology (GO) was performed.

### Quantitative real-time PCR assays

Total RNA was extracted from frozen-thawed mouse testes as above. The RNA was used to synthesize cDNA using reverse transcription kits. TB Green™ Premix Ex Taq™ II (TakaRa Biotechnology, Dalian, China) was used for real-time PCR on a LightCycler 96 Real-Time System (Roche, Basel, Switzerland). The primers of target genes were synthesized by Genewiz (South Plainfield, NJ). The sequences of primers were as follow: PGK2 (forward) 5′-TGGGGTATTTGAATGGGATGC-3′, (reverse) 5′-GTGCTCACATGGCTGACCTTG-3′; Cfap69 (F) 5′-TTGGTGGAATGTTGCATTCTGTG-3′, (R) 5′-GTCAAAGAAAGGGTGTAAAACGC-3′; Lin28b (F) 5′-TGTGCGAGAAGAAGAGTCCAGG-3′, (R) 5′-CATGATGCTCTGACAGTAATGGC-3′; Igf2bp1 (F) 5′-CTCCCCACCCCTAAATGACCT-3′, (R) 5′-CGCCTAACCTAAGCAAGTGGAG-3′; β-actin (F) 5′-CAGAAGGAGATTACTGCTCTGGC-3′, (R) 5′-GTCAAAGAAAGGGTGTAAAACGC-3′. The relative abundances of mRNA sequences were calculated and normalized to mean β-actin mRNA levels.

### Western blot analysis

Aliquots of 50 µg of protein were loaded onto 10% sodium dodecyl sulphate-polyacrylamide gels for electrophoresis, and transferred to PVDF membranes. Membranes were blocked with skimmed milk for 1.5 h, and then primary antibodies against PGK2, Cfap69, Lin28b, Igf2bp1, GAPDH or β-actin were incubated overnight at 4 °C. The membranes were rinsed for three times and then incubated with the corresponding secondary antibodies. Finally, the blots were detected using an Odyssey infra-red imaging system (Thermo Fisher Scientific, Waltham, MA). The housekeeping protein GAPDH or β-actin was used to standardize the protein density.

### Statistical analysis

All data are expressed as the mean ± standard deviations from at least three independent experiments. One-way ANOVA and Fisher’s *post hoc* least significant difference test were used for statistical analysis. Mean differences at *p*< 0.05 were considered statistically significant.

## Results

### Effects of SBP on testicular weight index and sperm quality in busulfan-treated mice

**Figure 1.**
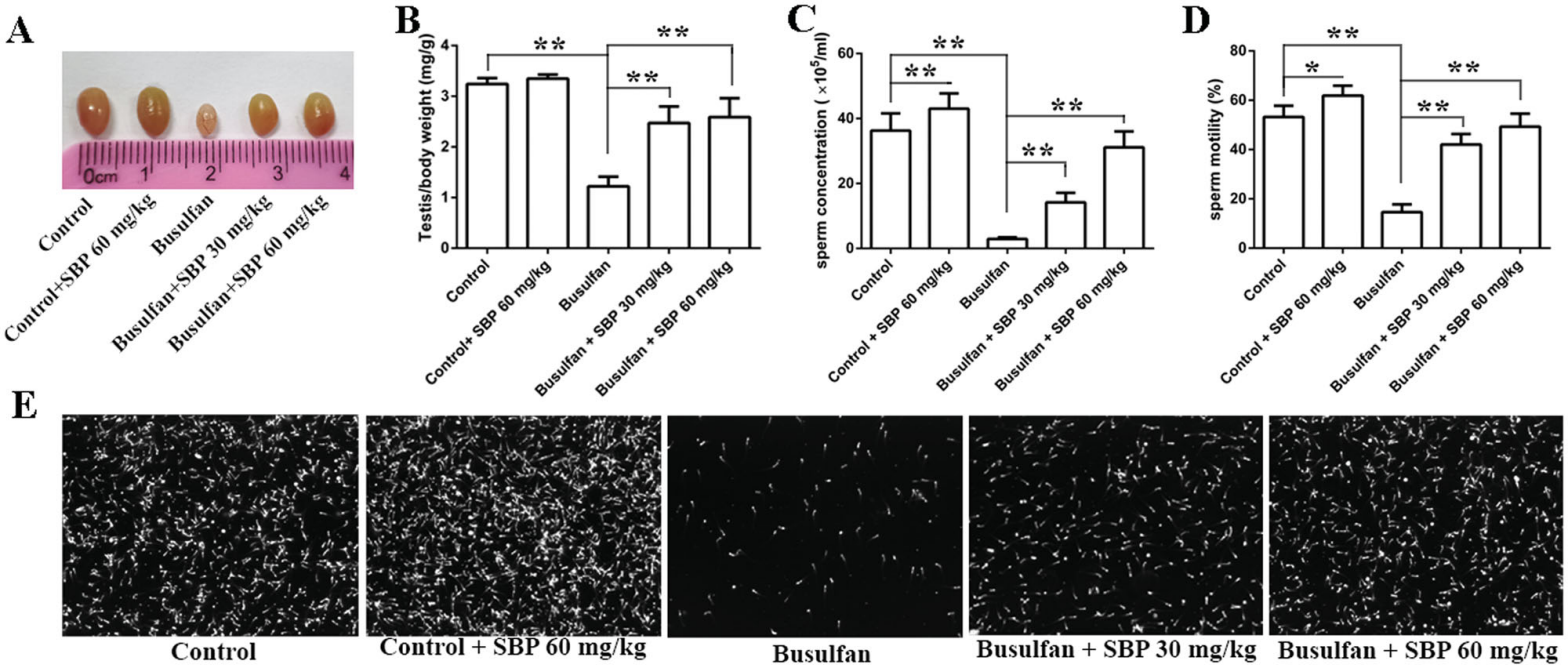
Swim bladder peptide treatment alleviated busulfan-induced reduction of sperm concentration and motility in mice. (A) Morphological observation of the testes in mice aged 8 weeks after treatment with Busulfan, Busulfan + SBP 30 mg/kg and Busulfan + SBP 60 mg/kg. (B) Testis weight index. (C) Epididymal sperm concentrations. (D) Epididymal sperm motility. (E) Morphology of sperms from epididymis. Data are expressed as mean ± SDE, with *n* = 6 for each group.

Compared with the control group, epididymal sperm concentration and motility were higher in the control + 60 mg/kg group (*p*< 0.05 or *p*< 0.01). However, there was no difference in testicular mass index between the two groups. Additionally, testicular mass index, epididymal sperm concentration and motility were lowered in the busulfan group than those in the control group (Figure 1). After treatment with SBP 30–60 mg/kg for 4 weeks, these indexes increased by 1.0–1.1-, 3.9–9.6- and 1.9–2.4-fold, respectively (*p*< 0.05 or *p*< 0.01).

### Effects of SBP on busulfan-induced damage to spermiogenesis

**Figure 2.**
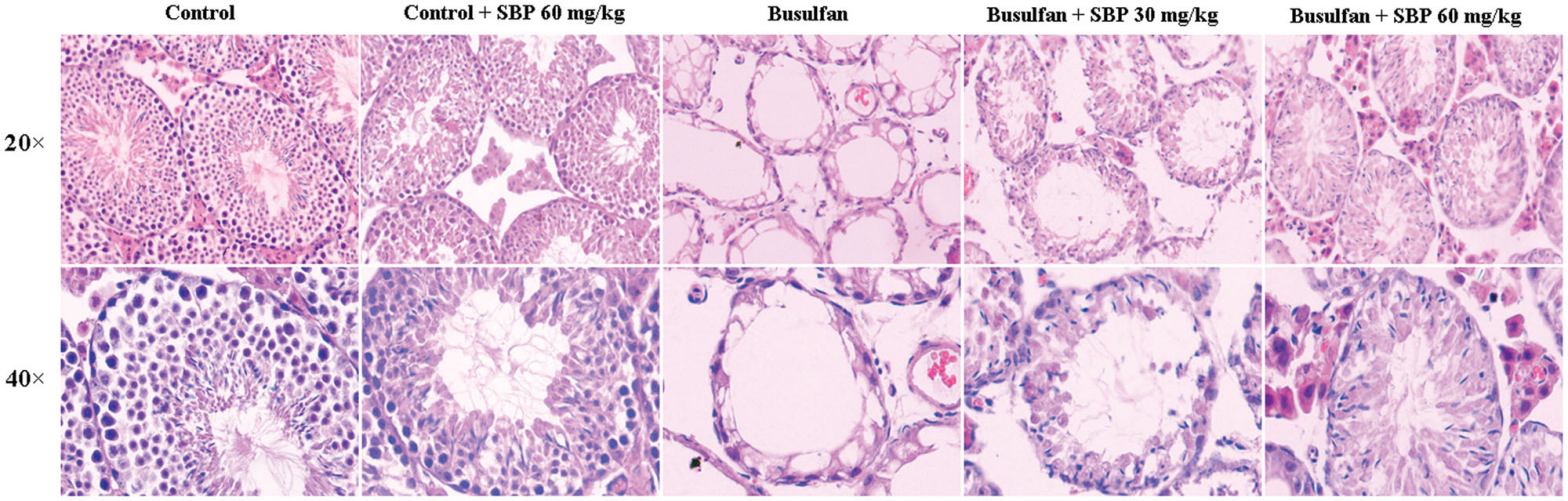
The representative seminiferous tubules architectures stained with haematoxylin–eosin (H&E) in each group (the upper row 20×; the below row 40×).

Histology showed that nearly all seminiferous tubules in the control and control + SBP 60 mg/kg groups were in complete spermatogenesis. After busulfan treatment, the numbers of spermatogenic cells, including spermatocytes, mature spermatozoa and spermatogonia were significantly decreased, leading to increased lumen diameters and vacuolization. These features were reversed by SBP treatment, especially in the 60 mg/kg group (Figure 2).

### Analysis of differentially expressed genes in testis tissues between busulfan and busulfan + SBP groups

**Figure 3.**
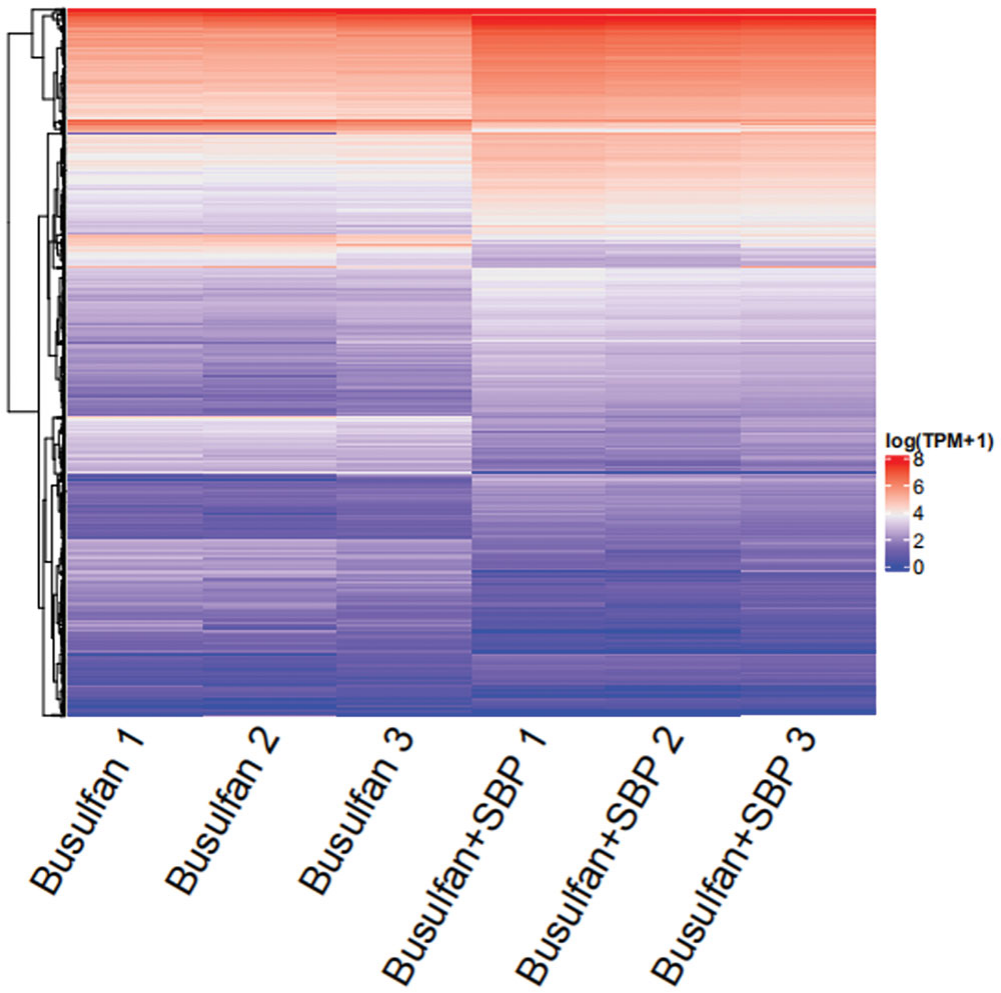
Heat map showing differentially expressed genes in testis tissue between mice in Busulfan and Busulfan + SBP groups. Low expression is depicted in blue, and high expression is depicted in red.

To explore the underlying mechanism of SBP on oligoasthenospermia, we used RNA sequencing analysis to determine which genes are differentially expressed in the testis between mice in busulfan and busulfan + SBP groups (Figure 3). After SBP treatment for 4 weeks, 561 genes were significantly up-regulated, while 1085 genes were down-regulated.

**Figure 4.**
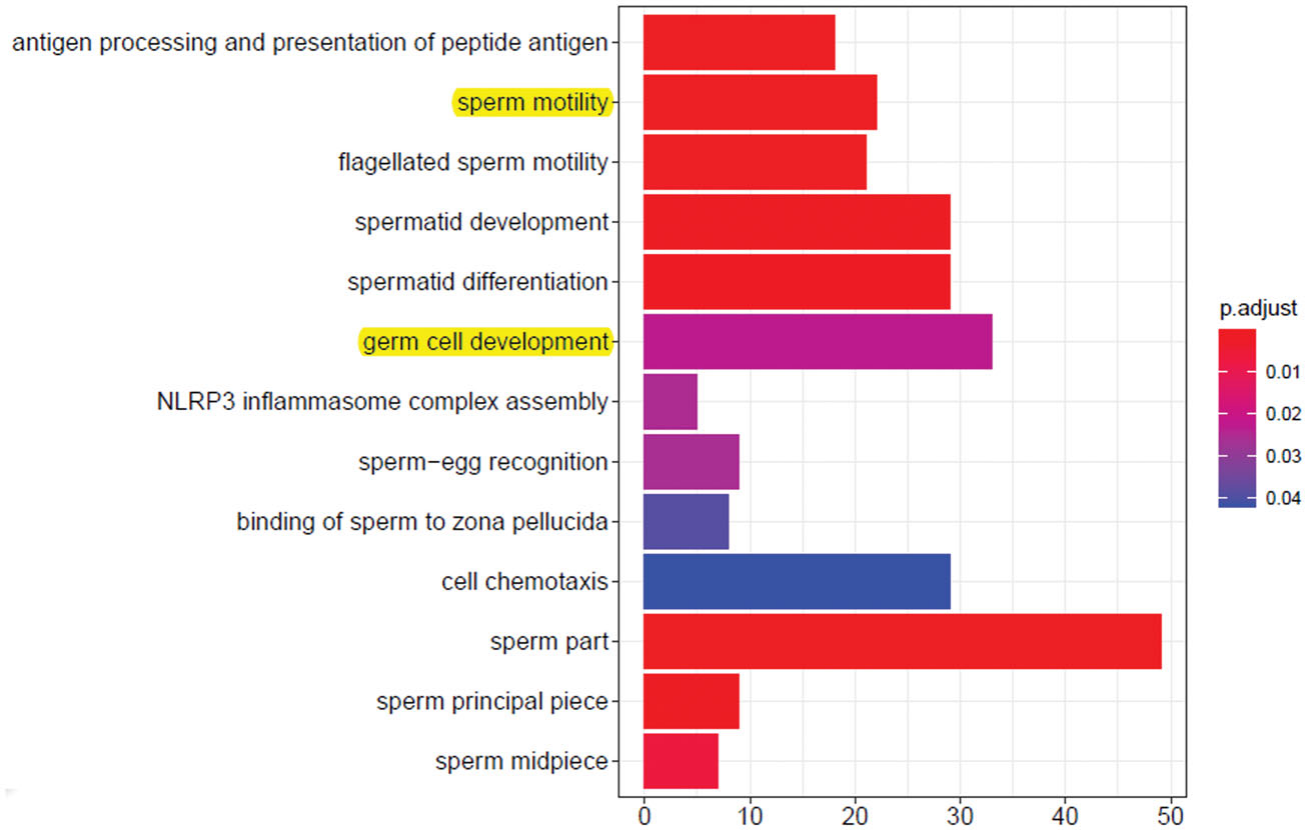
The top 13 most significant biological process terms in GO enrichment analysis. The *x*-axis represents the number of genes enriched in the corresponding biological process and the *y*-axis represents the GO classification.

In order to better understand the biological function of differentially expressed genes, we performed GO classification analysis based on RNA sequencing data. Figure 4 shows the top 13 GO enriched biological processes. Among the GO terms, ‘germ cell development’ and ‘sperm motility’ are closely related with the effect of SBP on spermatogenesis (Figure 4; yellow background). Among the differentially expressed mRNAs related to germ cell development, RNA-sequencing showed that, compared with the busulfan group, Lin28b and Igf2bp1 expression was remarkably increased after SBP treatment with a >1.5-fold change (*p*< 0.05). Meanwhile, among the differentially expressed mRNAs associated with sperm motility, the expression of PGK2 and Cfap69 was also significantly increased in the busulfan + SBP group with a >1.5-fold change that those in the busulfan group (*p*< 0.05).

### Verification of gene expression linked to germ cell development after SBP treatment

**Figure 5.**
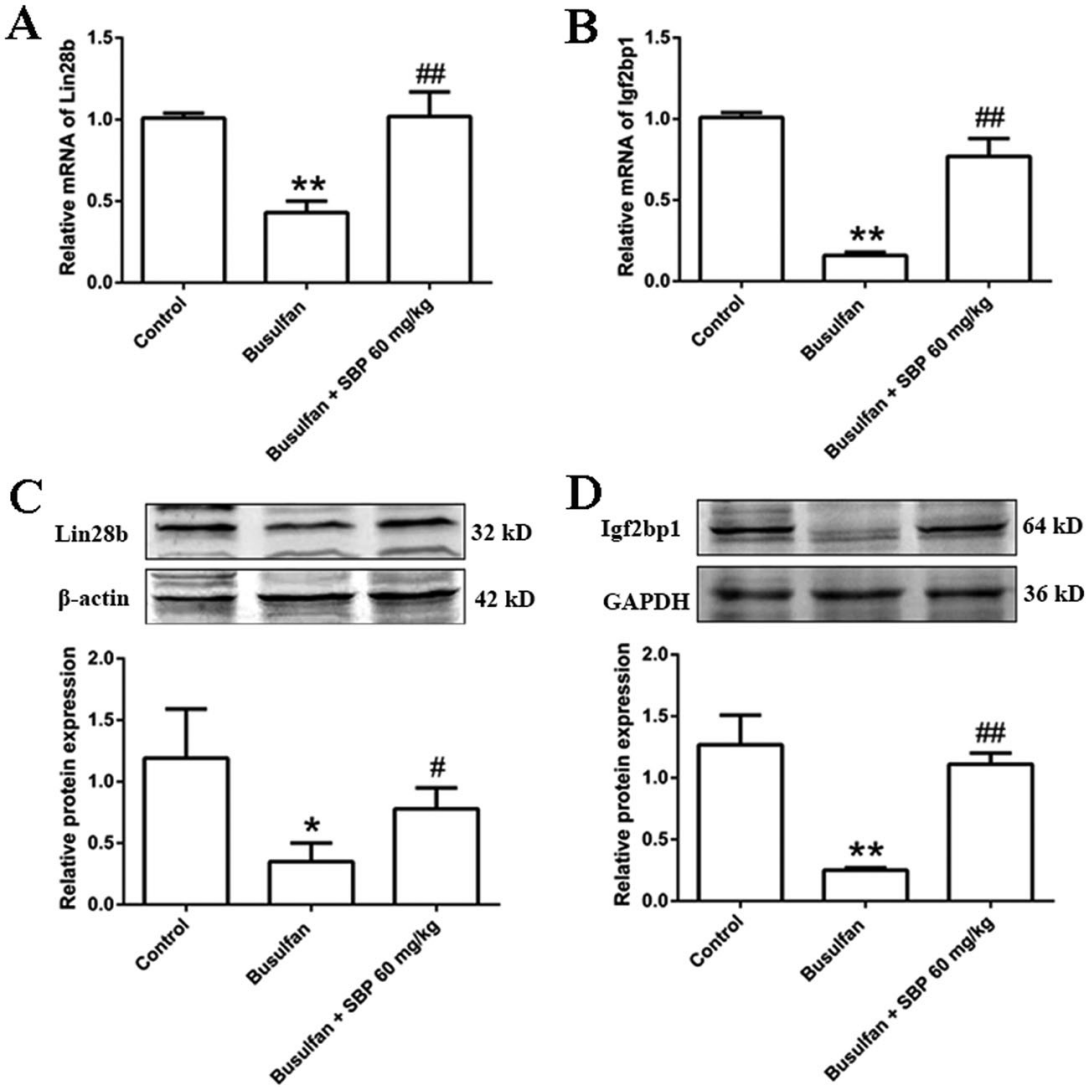
The effects of SBP on the expression of genes involved in germ cell development. (A) Relative Lin28b mRNA level. (B) Relative Igf2bp1 mRNA level. (C) The protein level of Lin28b and grayscale analysis. (D) The protein level of Igf2bp1 and grayscale analysis. *p< 0.05, **p< 0.01 vs. Control group; ^#^*p*< 0.05, ^##^*p*< 0.01 vs. Busulfan group. Data are expressed as mean ± SDE, with *n* = 3 for each group.

As is shown, the relative mRNAs levels of Lin28b and Igf2bp1 in the busulfan group were significantly lowered than those in the control group. However, Lin28b and Igf2bp1 mRNA levels were obviously elevated after SBP treatment. RT-qPCR results were in line with the RNA sequencing data. Moreover, Western blot experiments showed that protein levels of Lin28b and Igf2bp1 were also elevated after SBP treatment (Figure 5).

### Verification of gene expression related to sperm motility after SBP treatment

**Figure 6.**
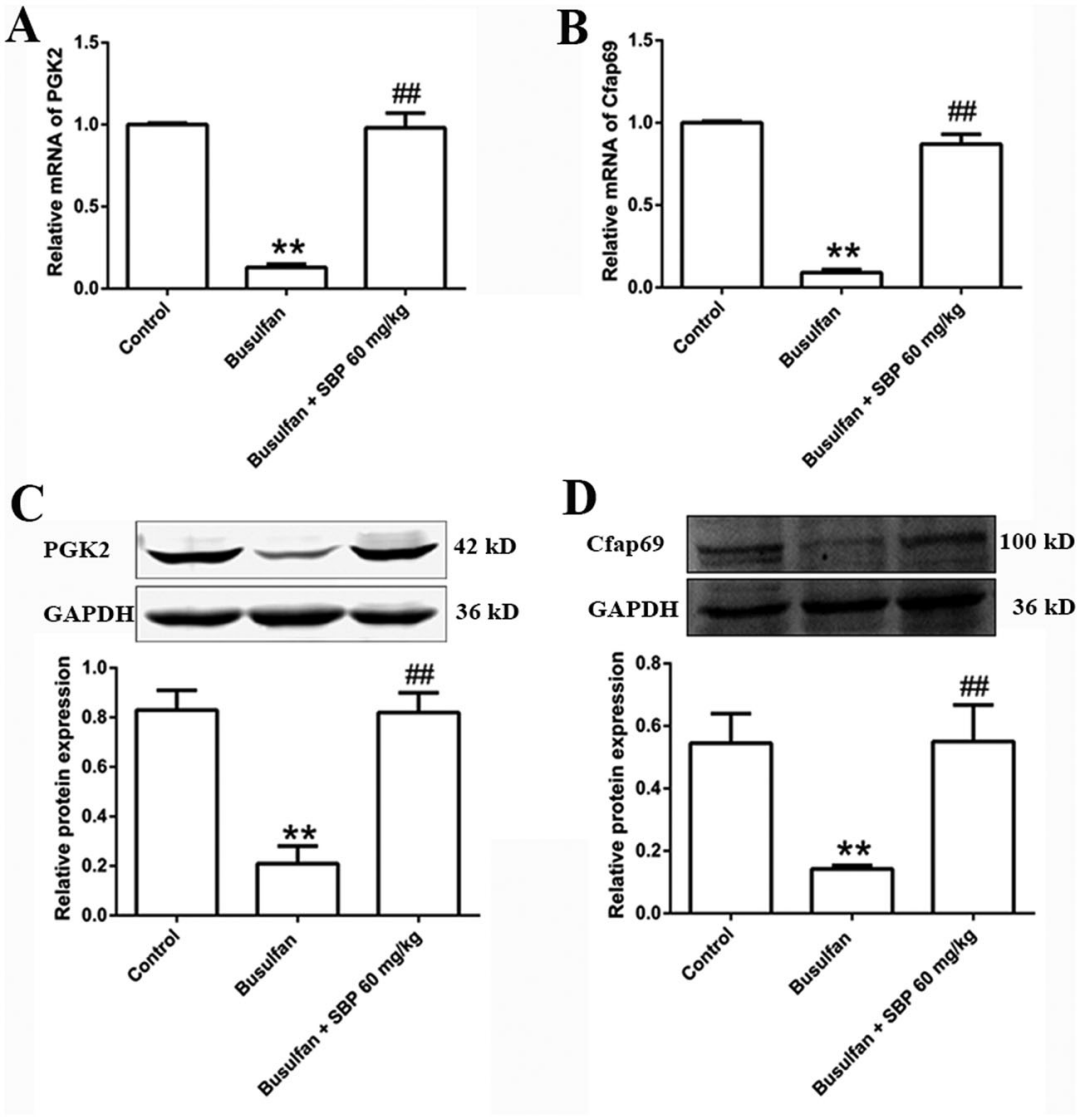
The effects of SBP on the expression of genes involved in sperm motility. (A) Relative PGK2 mRNA level. (B) Relative Cfap69 mRNA level. (C) The protein level of PGK2 and grayscale analysis. (D) The protein level of Cfap69 and grayscale analysis. **p< 0.01 vs. Control group; ^##^*p*< 0.01 vs. Busulfan group. Data are expressed as mean ± SDE, with *n* = 3 for each group.

Compared with the control group, the relative mRNA levels of PGK2 and Cfap69 were reduced dramatically in the busulfan group, while they were increased in the SBP treatment group. These results were consistent with the RNA-seq data. Subsequently, we detected protein levels of PGK2 and Cfap69 by Western blotting. Changes in the PGK2 and Cfap69 protein levels in every group were consistent with the mRNA levels (Figure 6).

## Discussion

Busulfan, a chemotherapeutic drug, is widely used to treat a variety of tumours to improve survival in patients. However, busulfan treatment may induce testicular tissue damage and male infertility (Chen et al. 2018; Nasimi et al. 2018). Therefore, strategies that effectively counteract the adverse reaction of busulfan on spermatogenesis to preserve fertility are required. Peptides from fish swim bladders have attracted considerable pharmaceutical attention thanks to their potential health benefits, high biological activity, water-solubility, easy absorption and non-toxicity (Agyei et al. 2016; Sila and Bougatef 2016).

Here, we extracted peptide from protein hydrolysate of croceine croaker swim bladder, and subsequently treated the mice with busulfan-induced oligoasthenospermia by using the SBP for 4 weeks. Treatment with SBP obviously elevated the decreased epididymal sperm concentration and motility caused by busulfan, and attenuated testicular damage and improved germ cell development. In order to investigate the underlying mechanism of the effect of SBP on oligoasthenospermia, we performed profiling studies. The RNA sequencing and GO enrichment analysis indicated that the biological processes of ‘germ cell development’ and ‘sperm motility’ were closely related with the effect of SBP on spermatogenesis. We choose Lin28b and Igf2bp1 from the differentially expressed mRNAs related to ‘germ cell development’, and PGK2 and Igf2bp1 from the differentially expressed mRNAs associated with ‘sperm motility’. RNA-sequencing showed that, compared with the busulfan group, the expressions of Lin28b, Igf2bp1, PGK2 and Cfap69 were remarkably increased after SBP treatment for 4 weeks.

Lin28b mRNA is expressed in various sarcomas but is not normally detectable in organs other than the testis. It is prominently expressed in spermatogonia and spermatocytes, and its activation can promote cell proliferation (Aeckerle et al. 2012; Sangiao-Alvarellos et al. 2015). Igf2bp1 is also expressed mainly in spermatogonia and plays a vital role in maintaining the pool of spermatogonial stem cells and in germ cell development (Hammer et al. 2005; Huang et al. 2018). It promotes cell proliferation via upregulation of Igf2bp1 (Chatterji et al. 2018; Zhang et al. 2020). Here, SBP treatment obviously upregulated Lin28b and Igf2bp1 mRNA and protein levels, so we infer that SBP promotes germ cell proliferation via upregulation of Lin28b expression, and subsequent modulation of the downstream protein Igf2bp1.

PGK2 is exclusively expressed in post-meiotic germ cells and is involved in glycolysis in spermatozoa, which is vital for sperm motility and male fertility. Previous studies revealed that PGK2 expression in spermatozoa from asthenozoospermia was obviously decreased, and sperm motility was markedly reduced in males lacking it (Danshina et al. 2010; Liu et al. 2016, 2019). In addition, Cfap69 is required for the assembly and stability of sperm flagellum. Cfap69 gene loss in mice caused a significant decrease in sperm motility from abnormalities of the sperm flagella (Dong et al. 2018; He et al. 2019; Mbango et al. 2019). Our results also showed that SBP treatment significantly increased PGK2 and Cfap69 mRNA and protein levels, indicating that SBP increases sperm motility mainly via upregulation of PGK2 and Cfap69 expression levels.

## Conclusions

Here we show, for the first time, evidence for the beneficial therapeutic effect of SBP on busulfan-induced oligoasthenospermia in mice. These effects appear to be mediated mainly by upregulating the protein levels of Lin28b and Igf2bp1 to promote germ cell proliferation to increase sperm concentration, and by upregulating PGK2 and Cfap69 to increase sperm motility. We have applied for a patent for SBP in the treatment of oligoasthenospermia. In the further study, we will investigate the half-life period, the safe dose range, and the effective therapy dose of SBP in humans, which will provide experimental basis for SBP in the treatment of male infertility.
